# Mendelian Randomization Study on Serum Metabolites and Diabetic Nephropathy Risk: Identifying Potential Biomarkers for Early Intervention

**DOI:** 10.2174/0113816128377862250429045226

**Published:** 2025-05-12

**Authors:** Siyuan Song, Jiangyi Yu

**Affiliations:** 1 Jiangsu Province Hospital of Chinese Medicine, Affiliated Hospital of Nanjing University of Chinese Medicine, Nanjing, China

**Keywords:** Serum metabolites, diabetic nephropathy, mendelian randomization, instrumental variables, type 2 diabetes mellitus, metabolism

## Abstract

**Objective:**

In this study, the causation between serum metabolites and the risk of Diabetic Nephropathy (DN) was investigated by means of a Mendelian Randomization (MR) analysis.

**Methods:**

Our data on diabetic nephropathy were obtained from the IEU OpenGWAS Project database, while serum metabolite data originated came from the GWAS summary statistics by Chen *et al*. The Inverse Variance Weighted (IVW) method was the main analysis approach, with Weighted Median (WME) and MR-Egger regression serving as supplementary approaches to construing the causalities between serum metabolites and the DN risk. In addition to the MR-Egger regression intercept, Cochran's Q test was utilized for sensitivity analysis, with *P* values used as the metric to assess the results.

**Results:**

In total, 14 SNPs regarding serum metabolites were chosen as Instrumental Variables (IVs). The IVW results indicated that levels of Behenoylcarnitine (C22), Arachidoylcarnitine (C20), and the ratio of 5-methylthioadenosine (MTA) to phosphate exerted a positive causal effect on the DN risk. Conversely, levels of 5-hydroxylysine, Butyrylglycine, 1-stearoyl-glycerophosphocholine (18:0), Isobutyrylglycine, 1-stearoyl-2-oleoyl-GPE (18:0/18:1), N2,N5-diacetylornithine, 2-butenoylglycine, 3-hydroxybutyroylglycine, N-acetyl-isoputreanine, the ratio of Arginine to Ornithine, and the ratio of Aspartate to Mannose exerted a negative impact of causality on the DN risk. By identifying these serum metabolites, high-risk patients can be recognized in the early stages of diabetic nephropathy, enabling preventive measures or delaying its progression. These findings also provide a solid foundation for further research into the underlying etiology of diabetic nephropathy.

**Conclusion:**

The translation of serum metabolites into clinical applications for DN aims to utilize changes in serum metabolites as biomarkers for early diagnosis, thereby monitoring the progression of DN and providing a foundation for personalized treatment. For instance, the development of serum metabolite diagnostic kits could be used for early detection and prevention of DN. Changes in metabolites can help identify different stages of DN.

## 1 INTRODUCTION

Diabetic Nephropathy (DN) has become a typical complication of type 2 diabetes mellitus (T2DM) [1]. Although multiple studies have demonstrated that Renin-Angiotensin System Inhibitors (RASis), Sodium-Glucose Cotransporter-2 Inhibitors (SGLT-2is), and novel Mineralocorticoid Receptor Antagonists (MRAs) significantly reduce renal composite endpoints along with their widespread clinical adoption as recommended by various guidelines, the incidence of DKD and its progression to End-Stage Kidney Disease (ESKD) continue to rise [2-6]. The interplay between inflammatory cytokines and factors like the endocrine and immune systems, oxidative stress, and abnormal fat metabolism correlates with changes in cellular components, endoplasmic reticulum stress [7], ferroptosis [8], microvascular disease, and insulin resistance [9]. Changes in metabolites have been significantly correlated with the progression of DN [10]. Wei [11] observed that the metabolites of lactic acid, allantoin, and acetic acid initially increased and then decreased during the development of DN, suggesting that metabolite changes may represent a dynamic process. In a state of high glucose, excess glucose cannot enter cells effectively due to insufficient insulin, leading to disrupted glucose metabolism and consequently affecting lipid and protein metabolism. Zhao [12] found that sorbitol, fructose, and N-acetylglucosamine levels increased significantly in the kidneys of DN rats, with these metabolites participating in the polyol and hexosamine pathways, which are closely linked to DN pathogenesis [13]. Taurine, an effective endogenous antioxidant, has demonstrated defensive influences against STZ-induced renal lesions among diabetic rats [14]. Research indicates that serum metabolites can differentiate DN patients with significant albuminuria from those without it [15]. Nonetheless, observational research is often affected by unclear confounding factors and reverse causation, making the causal relationship between serum metabolites and DN risk uncertain.

Mendelian Randomization (MR) acts as a valid approach to inferring causalities between exposure and outcome through single nucleotide polymorphisms (SNPs) as instrumental variables (IVs) [16, 17]. Recently, MR has been progressively popular as an alternative method of assessing causalities [18]. In contrast to traditional observational studies, MR offers several distinct advantages, particularly in overcoming biases and confounding factors, thereby enabling a more accurate elucidation of causal relationships [19]. Observational studies are often susceptible to a range of potential biases, including selection bias, reporting bias, and recall bias, which can substantially distort the findings. Moreover, controlling for all potential confounders remains a challenging task, as even after multivariable adjustment, unmeasured or difficult-to-quantify confounders may still influence the interpretation of results [20]. By leveraging genetic variants as instrumental variables, the MR method effectively mitigates these biases, thereby providing more robust causal evidence. This approach is particularly valuable when observational findings are ambiguous or conflicting, as MR serves to address these limitations and strengthen the foundation for causal inference [21]. In this study, data were collected through the Genome-Wide Association Study (GWAS). An MR analysis was performed to investigate the causalities between serum metabolites and the DN risk, providing genetic support for their correlation. Fig. (**1**) displays our research protocol.

## 2 MATERIALS AND METHODS

### 2.1 Exposure and Outcome Data Sources

GWAS represents a powerful approach for identifying associations between genetic variations and complex traits across the entire genome. This method is primarily employed to uncover the genetic underpinnings of complex traits and to identify genetic loci associated with diseases [22]. SNPs refer to DNA sequence variations at the genomic level caused by a single nucleotide change. These variations are pervasive throughout the human genome. SNP polymorphisms typically involve alterations at a single base pair, which can result from transitions (substitutions between purines or pyrimidines), transversions (substitutions between a purine and a pyrimidine), or insertions or deletions of bases [23].

In terms of effect estimations for SNPs regarding serum metabolites, GWAS summary statistics from Chen *et al*. (GCST90199621-GCST902010202) were employed, which implemented genome-wide genotyping and evaluated circulatory plasma metabolites in 8,299 unrelated European people. Complying with the rigorous pre-GWAS genotype quality control (QC), about 15.4 million SNPs were applied to the analysis. Among plasma samples, the levels of 1,458 metabolites were quantified through the Ultrahigh Performance Liquid Chromatography-Tandem Mass Spectrometry (UPLC-MS/MS) platform. Following normalization involving the removal of substances regarding system artifacts, background noises, and bad allocations, an entire QC process identified 1,091 metabolites comprising 850 identified substances and 241 unidentified substances with 309 metabolite proportions for inclusion in genome-wide association studies [24]. The research data (ebi-a-GCST90018832) [25] were derived from the IEU OpenGWAS Project site. The capacity of the DN dataset samples reached 452,280, which included 451,248 controls, and the number of SNPs reached 24,190,738. In this study, we selected the samples derived from the European population, which helped reduce external confounding factors in genotype and phenotype data. A homogeneous population ensures more accurate estimates of the genetic-disease associations, as this similarity minimizes the impact of environmental factors on the study outcomes, thereby enhancing the accuracy and reliability of the findings [26]. Due to utilizing public data, extra consent or ethical approval was not necessary for our research.

### 2.2 Instrumental Variable Selection

The gene variations chosen as IVs were associated with serum metabolites. The study design was based on the following criteria [27]: (1) SNPs were significantly associated with the exposure of interest (association assumption); (2) SNPs were independent of any confounding factors (independence assumption); (3) SNPs influenced the outcome solely through the exposure, with no pleiotropy (exclusivity assumption). Effective IVs were selected based on these assumptions. Initially, SNPs were extracted from GWAS data, and inclusion criteria were set at *P* < 5e-08. To elude the influence of linkage disequilibrium (LD) on research findings, SNPs were filtered to ensure R2 < 0.001 and a distance of at least 10,000 kb between them [28]. The statistical power of SNPs was determined by their predictive ability for the phenotype (R2), which was appraised *via* the relationship strength between SNPs and the phenotype. R2 indicated the ratio of variation in the exposure interpreted by the genetic variant, indicating the strength of the genetic instrument. Higher R2 values suggested a stronger instrument [29].

Furthermore, the PhenoScanner database was applied to deeply corroborate if the aforementioned SNP loci correlated with the remaining confounders [30]. Ultimately, to appraise if the included SNPs were influenced by weak IVs, *F* statistics were employed to remove *F* values exceeding 10 (the formula was below *F* = *β*^2^/*SE*^2^. Here, *β* indicates the value of the allele effect with *SE* indicating the standard error). If the *F*-statistic of the SNPs fell short of 10, it meant that the SNPs had the likelihood of weak IV bias and were subsequently removed to avoid interfering with the outcomes [31].

### 2.3 Statistical Analysis

#### 2.3.1 MR Analysis

Inverse Variance Weighted (IVW), MR-Egger regression, Weighted Median (WME), and forest plots were applied to construe the causalities between serum metabolites and DN risks, ensuring the credibility of results [32]. The IVW method was the primary approach used for MR analysis. Without the horizontal pleiotropy, IVW provided a stable and accurate assessment of the cause and effect of exposure on the outcome through meta-analysis integrated into the Wald estimate for each IV [33]. The IV did not exhibit horizontal pleiotropy, making it the most powerful method for testing in all analyses [34]. MR-Egger regression was used for checking the oriented pleiotropy [35], which can detect and accommodate pleiotropy in instrument variables. When genetic pleiotropy is present, it provides less biased effect estimates compared to IVW [36]. According to heterogeneity and pleiotropy analyses, the final MR method was chosen. If heterogeneity was present but there was no pleiotropic effect, IVW integrated with the Multiplicative Random Effect (IVW-MRE) was employed. The advantage of the IVW-MRE method lies in its ability to provide high estimation precision when instrument variables do not exhibit pleiotropy. This approach integrates the effect estimates of various genotype combinations through inverse variance weighting, ensuring an accurate overall causal effect estimate. Even in the presence of pleiotropy in the instrument variables, it can still provide unbiased estimates [37]. In the presence of pleiotropy, with or without heterogeneity, MR-Egger regression was again utilized [38]. WME determined the median of the distribution function, which was derived by ranking the SNP effect values of all individuals according to their weights. Since at least 50% of the information was derived from valid IVs, WME provided a robust estimate [39]. Causality between serum metabolites (exposure) and DN (outcome) was established when *P* < 0.05 [40].

#### 2.3.2 Sensitivity Analysis

From Cochran's Q statistic for IVW, the *P* value was employed to examine the heterogeneity of IVs. As *P* ≥ 0.05, no heterogeneity in causality analysis emerged [35]. Furthermore, a funnel plot was used for checking the heterogeneity, and symmetric SNP distribution denoted no heterogeneity of the outcomes [41]. Pleiotropy was rated through MR-Egger regression, with the interception construed in a scatter plot [42]. To appraise the effect of each SNP, a Leave-one-out (LOO) analysis was performed [43]. The MR-Egger interception experiment measured the effect of pleiotropy on cause and effect, with the pleiotropy illustrated through a nonzero interception. Among the IVa, the heterogeneity was rated through Cochran's Q test. The LOO method investigated the effect of each IV and the robustness of results by removing an IV at a time and gauging the group effect of the rest of the IVs [44]. The risk correlation between serum metabolites and DN was denoted as an odds ratio (*OR*), showing a 95% Confidence Interval (*CI*). More importantly, *P* < 0.05 reached statistical significance.

#### 2.3.3 Enrichment Analysis

SNPs, which were demonstrated to have a significant causality with DN based on the screening criteria established in this study, were imported into the G. Profiler site and converted into Gene IDs. Kyoto Encyclopedia of Genes and Genomes (KEGG) enrichment analyses were carried out through the DAVID website, with the top 10 enrichment results visualized using R software.

#### 2.3.4 Statistical Software

The total MR analyses were executed through the TwoSampleMR package and R (v 4.3.1).

## 3 RESULTS

### 3.1 Instrumental Variable Selection

**Fig. (2):**
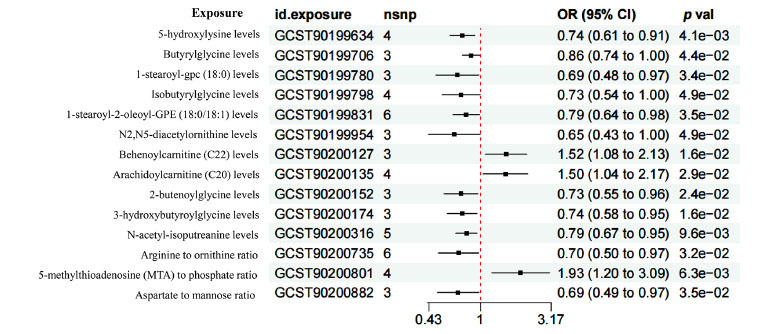
Forest plot of the MR results of 14 serum metabolites. Exposure signifies the serum metabolites, nSNP signifies the number of SNPs, and pval signifies the *p*-value.

In accordance with the screening criteria for IVs, a total of 4, 3, 3, 4, 6, 3, 3, 4, 3, 3, 5, 6, 4, and 3 SNPs were extracted from the following metabolites: of 5-hydroxylysine, butyrylglycine,1-stearoyl-glycerophosphocholine (18:0), isobutyrylglycine, 1-stearoyl-2-oleoyl-GPE (18:0/18:1), N2,N5-diacetylornithine, behenoylcarnitine (C22), arachidoylcarnitine (C20), 2-butenoyl-glycine, 3-hydroxybutyroylglycine, and N-acetyl-isoputreanine, along with the the ratio of arginine to ornithine, the ratio of 5-methylthioadenosine (MTA) to phosphate, and the ratio of aspartate to mannose (Fig. **2**). For all included IVs, the *F* statistics exceeded 10, which indicated that the influence of weak IVs on the results would be minimal (Table **S1**).

### 3.2 MR Analysis

**Fig. (3):**
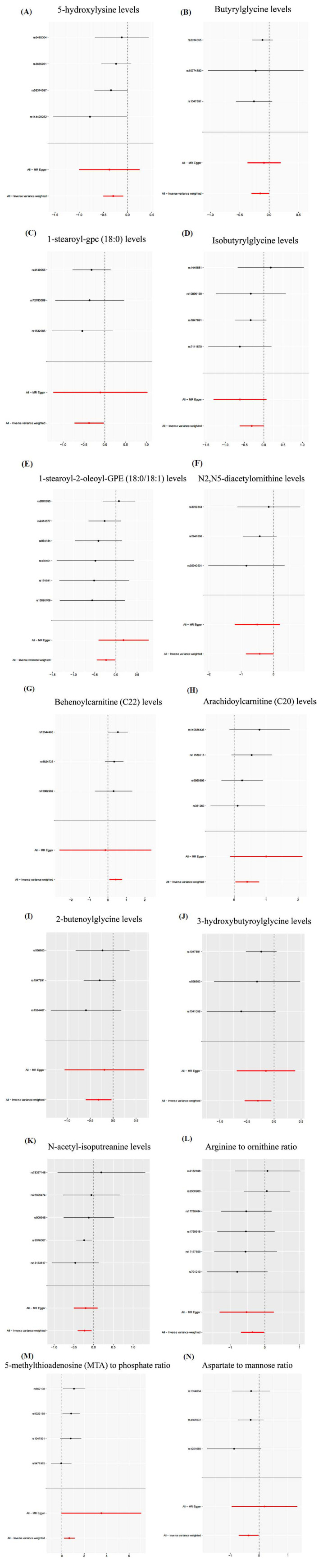
Forest plot of single SNP MR results. (**A**) 5-hydroxylysine levels; (**B**) Butyrylglycine levels; (**C**) 1-stearoyl-gpc (18:0) levels; (**D**) Isobutyrylglycine levels; (**E**) 1-stearoyl-2-oleoyl-GPE (18:0/18:1) levels; (**F**) N2,N5-diacetylornithine levels; (**G**) Behenoylcarnitine (C22) levels; (**H**) Arachidoylcarnitine (C20) levels; (**I**) 2-butenoylglycine levels; (**J**) 3-hydroxybutyroylglycine levels; (**K**) N-acetyl-isoputreanine levels; (**L**) Arginine to ornithine ratio; (**M**) 5-methylthioadenosine (MTA) to phosphate ratio; (**N**) Aspartate to mannose ratio; Here, each black dot signifies DN, showing a heightened Standard Deviation (SD) among serum metabolites derived from treating each SNP as an instrumental variable (IV). In contrast, the red dot signifies the causality estimation from the whole SNP combinations through multiple MR methods. The segment of the horizontal line indicates 95% CI. Notably, the IVW causality estimation demonstrates how the comprehensive estimation (illustrated by the red horizontal line) may be disproportionally impacted by excluding a single variant (represented by the black horizontal line).

**Fig. (4):**
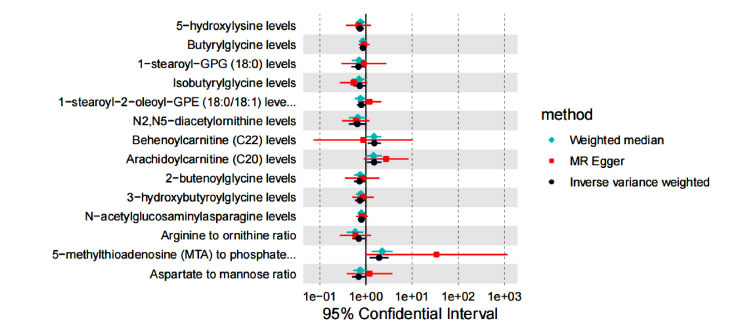
Forest plot of MR estimates of the causal effects of 14 serum metabolites on DN. The green diamond indicates the WME method, the red square indicates the MR Egger method and the black circle indicates the IVW method.

IVW analysis illustrated that the levels of behenoylcarnitine (C22) (*OR* =1.518, 95%*CI* 1.083-2.129, *P* = 0.016), the levels of arachidoylcarnitine (C20) (*OR* =1.503, 95% *CI* 1.042-2.168, *P* = 0.029), and the ratio of 5-methylthioadenosine (MTA) to phosphate (*OR* =1.930, 95% *CI* 1.204-3.093, *P* = 0.006) had a causality with the heightened risk of DN, whereas the levels of 5-hydroxylysine (*OR* =0.743, 95% *CI* 0.606-0.910, *P* = 0.004), butyrylglycine (*OR* =0.859, 95% *CI* 0.741-0.996, *P* = 0.044), 1-stearoyl-gpc (18:0) (*OR* =0.685, 95% *CI* 0.483-0.971, *P* = 0.034), isobutyrylglycine (*OR* =0.731, 95% *CI* 0.536-0.999, *P* = 0.049), 1-stearoyl-2-oleoyl-GPE (18:0/18:1) (*OR* =0.793, 95% *CI* 0.639-0.984, *P* = 0.035), N2,N5-diacetylornithine (*OR* =0.652, 95% *CI* 0.426-0.997, *P* = 0.049), 2-butenoylglycine (*OR* =0.727, 95% *CI* 0.550-0.959, *P* = 0.024), 3-hydroxybutyroylglycine (*OR* =0.741, 95% *CI* 0.581-0.946, *P* = 0.016), and N-acetyl-isoputreanine (*OR* =0.795, 95% *CI* 0.668-0.946, *P* = 0.010), along with the the ratio of arginine to ornithine (*OR* =0.699, 95% *CI* 0.503-0.969, *P* = 0.032), and the ratio of Aspartate to mannose (*OR* =0.695, 95% *CI* 0.495-0.975, *P* = 0.035) possessed a causality with the decreased DN risk (Figs. **3A**-**N**). Though the results of the WME and MR-Egger regression reached no statistical significance, the result directions corresponded to those of the IVW analysis (Table **S2**, Fig. **4**). Given that the IVW results were the most robust and conformed to the forest plot, serum metabolites were proven to correlate with the occurrence of DN. 

We hypothesize that elevated levels of behenoylcarnitine (C22), arachidoylcarnitine (C20), and the ratio of 5-methylthioadenosine (MTA) to phosphate may contribute to the onset and progression of DN by enhancing oxidative stress through mitochondrial dysfunction. Mitochondrial dysfunction-induced oxidative stress exacerbates cellular damage, promotes inflammation, and contributes to glomerulosclerosis, all of which are key factors in the pathological progression of DN [45, 46].

### 3.3 Sensitivity Analysis

**Fig. (5):**
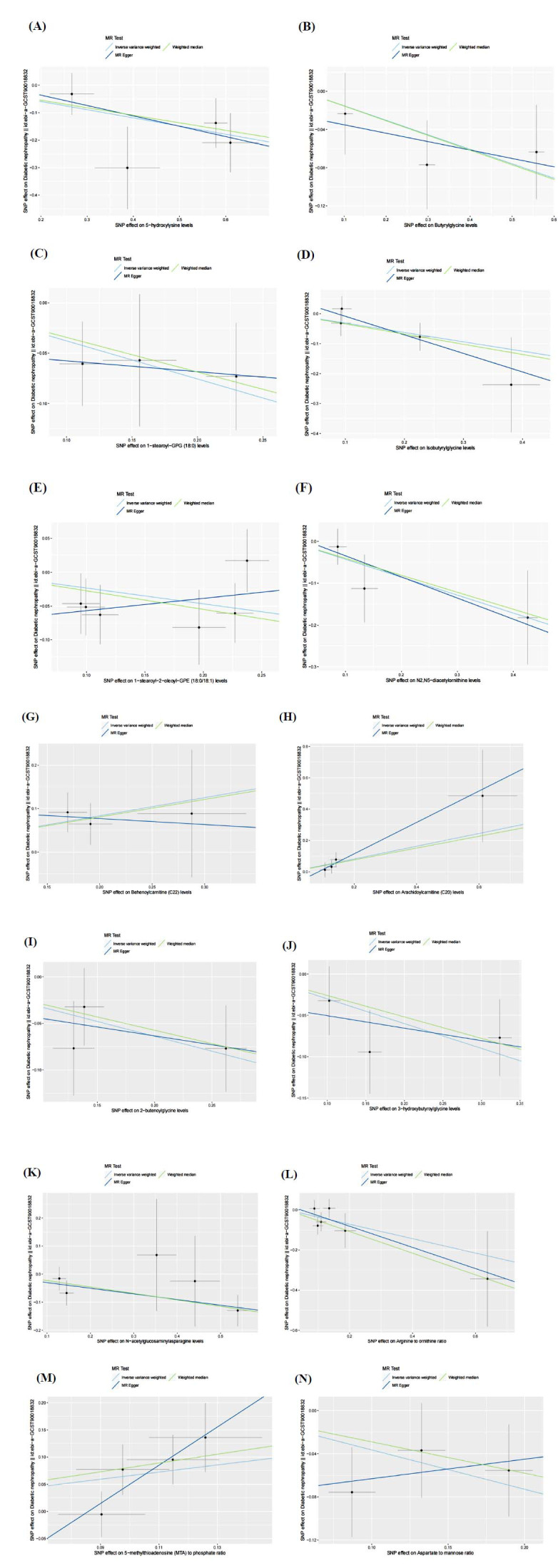
Scatter plots of SNP analysis. (**A**) The levels of 5-hydroxylysine; (**B**) The levels of Butyrylglycine; (**C**) The levels of 1-stearoyl-gpc (18:0); (**D**) The levels of Isobutyrylglycine; (**E**) The levels of 1-stearoyl-2-oleoyl-GPE (18:0/18:1); (**F**) The levels of N2, N5-diacetylornithine; (**G**) The levels of Behenoylcarnitine (C22); (**H**) The levels of Arachidoylcarnitine (C20); (**I**) The levels of 2-butenoylglycine; (**J**) The levels of 3-hydroxybutyroylglycine; (**K**) The levels of N-acetylglucosaminylasparagine; (**L**) The ratio of Arginine to ornithine; (**M**) The ratio of 5-methylthioadenosine (MTA) to phosphate; (**N**) The ratio of Aspartate to mannose; The X-axis indicates the effect of SNPs on serum metabolites, whereas the Y-axis depicts the effect of SNPs on DN. Each black dot corresponds to a single SNP, and the line segment indicates 95% CI. The straight line slope indicates the causality estimate from the MR method. The light blue line represents the IVW estimate, the blue one indicates the MR Egger estimation, and the green one indicates the WME estimation.

**Fig. (6):**
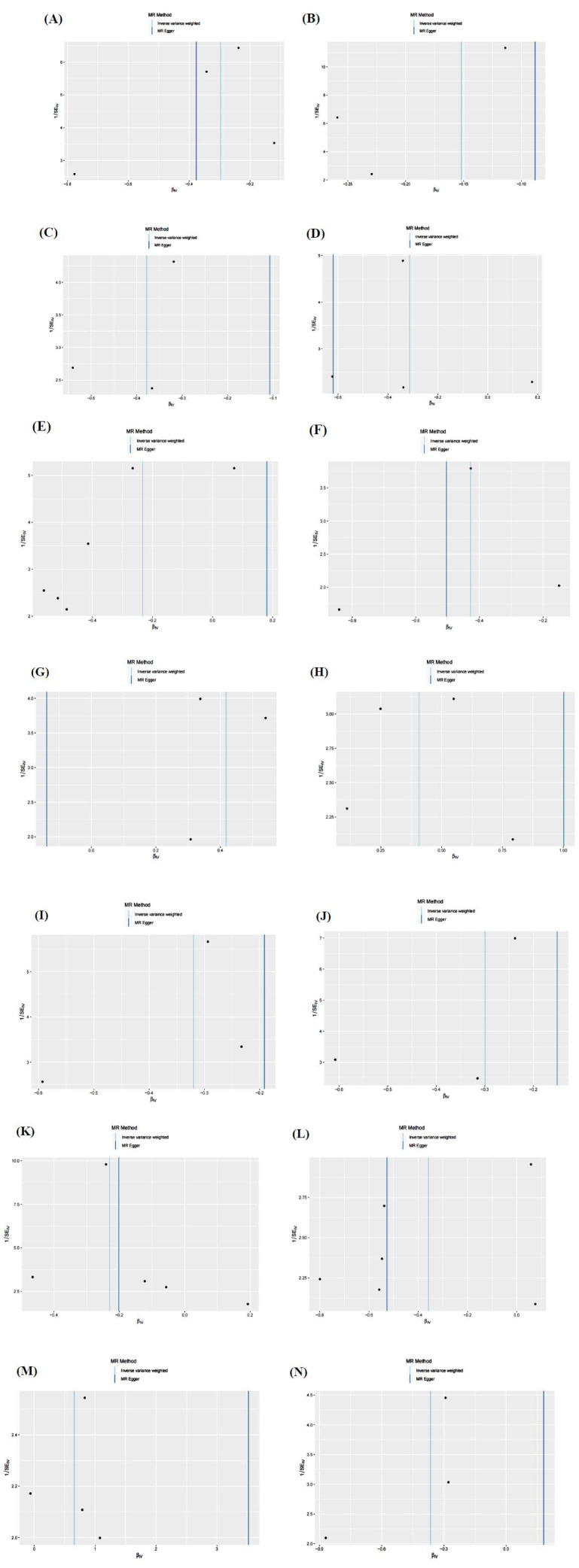
Funnel plots of sensitivity analysis. (**A**) 5-hydroxylysine levels; (**B**) Butyrylglycine levels; (**C**) The levels of 1-stearoyl-gpc (18:0); (**D**) The levels of Isobutyrylglycine; (**E**) The levels of 1-stearoyl-2-oleoyl-GPE (18:0/18:1); (**F**) N2,N5-diacetylornithine levels; (**G**) Behenoylcarnitine (C22) levels; (**H**) Arachidoylcarnitine (C20) levels; (**I**) 2-butenoylglycine levels; (**J**) 3-hydroxybutyroylglycine levels; (**K**) N-acetyl-isoputreanine levels; (**L**) Arginine to ornithine ratio; (**M**) 5-methylthioadenosine (MTA) to phosphate ratio; (**N**) Aspartate to mannose ratio; The light blue indicates the IVW method, with the dark blue indicating the MR Egger method.

**Fig. (7):**
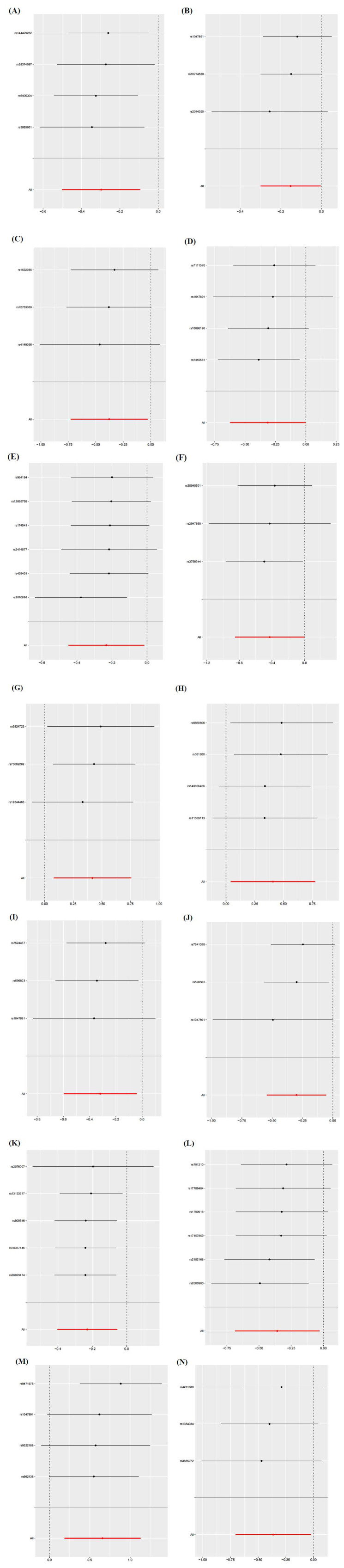
Forest plots of LOO sensitivity analysis. (**A**) 5-hydroxylysine levels; (**B**) Butyrylglycine levels; (**C**) The levels of 1-stearoyl-gpc (18:0); (**D**) The levels of Isobutyrylglycine; (**E**) The levels of 1-stearoyl-2-oleoyl-GPE (18:0/18:1); (**F**) N2,N5-diacetylornithine levels; (**G**) Behenoylcarnitine (C22) levels; (**H**) Arachidoylcarnitine (C20) levels; (**I**) 2-butenoylglycine levels; (**J**) 3-hydroxybutyroylglycine levels; (**K**) N-acetyl-isoputreanine levels; (**L**) Arginine to ornithine ratio; (**M**) 5-methylthioadenosine (MTA) to phosphate ratio; (**N**) Aspartate to mannose ratio; Here, each black dot signifies the DN, showing heightened SD in serum metabolites produced by employing each SNP as an IV. The red dot signifies the causality estimate from the whole SNP combinations through multiple MR methods. The segment of the horizontal line signifies 95% CI. To be specific, the IVW causal estimation manifests how the overall estimation (illuminated by the red horizontal line) may be affected disproportionally by removing a single variant (illuminated by the black horizontal line).

According to Cochran's Q test, the IVs revealed no heterogeneity (*P* > 0.05). The interception experiment of MR-Egger regression suggested that pleiotropy did not affect the results (*P* > 0.05) (Table **S3**, Figs. **5A**-**N**). Funnel plots further demonstrated that underlying confounders were less likely to influence the causation (Figs. **6A**-**N**). Leave-one-out (LOO) sensitivity analysis revealed that the removal of individual SNPs, in turn, exerted no remarkable influence on analysis results (Figs. **7A**-**N**).

### 3.4 Enrichment Analysis

The identified SNPs, which had a significant causality with DN, were converted into Gene IDs (Table **S4**). In terms of MR analysis, the KEGG results were enriched using the DAVID databases, leading to ten beneficial pathways in Fig. (**8**).

The biosynthesis of arginine, unsaturated fatty acids, and amino acids, bile secretion, butanoate metabolism, beta-alanine metabolism, fatty acid degradation, lysine degradation, valine, leucine and isoleucine degradation, as well as the PPAR signaling pathway, may be underlying factors contributing to the onset and progression of diabetic nephropathy DN.

## 4 DISCUSSION

Our research employed large-scale GWAS data, with an MR method employed to construe the causality between serum metabolites and DN. The IVW outcomes denoted that the levels of behenoylcarnitine (C22), arachidoylcarnitine (C20), and the ratio of 5-methylthioadenosine (MTA) to phosphate had a positive causal effect on the DN risk. Conversely, the levels of 5-hydroxylysine, butyrylglycine, 1-stearoyl-glycerophosphocholine (18:0), isobutyrylglycine, 1-stearoyl-2-oleoyl-GPE (18:0/18:1), N2, N5-diacetyl ornithine, 2-butenoylglycine, 3-hydroxybutyroylglycine, and N-acetyl-isoputreanine along with the ratio of arginine to ornithine and the ratio of aspartate to mannose had a causal negative effect on DN risk. Further sensitivity analysis confirmed that these results were consistent and reliable.

The imbalance of fatty acid metabolism was closely linked to kidney diseases, and fatty acid-related metabolic enzymes might serve as potential intervention targets for these conditions [47]. Pena found that levels of behenoylcarnitine were positively associated with urinary albumin excretion and negatively associated with changes in estimated glomerular filtration rate (eGFR) [48], potentially reflecting reduced mitochondrial oxidative capacity observed in patients with type 2 diabetes mellitus [49].

It has been confirmed that behenoylcarnitine concentration increases with progressive renal function loss. As claimed by Janos Kerner, the enrichment of (13)C in arachidoylcarnitine suggests that most of the two-carbon units applied to chain extension originate in the β-oxidation of [1,2,3,4-(13) C^4^] palmitic acid [50]. Methylthioadenosine (MTA) was an intermediate metabolite in the methionine (MET) cycle and polyamine synthesis, functioning to regulate gene expression and cellular proliferation [51]. Tan observed that 5-MTA expression increased in patients with diabetic sarcopenia [52]. 5-Hydroxylysine was found in collagen in a glycosylated form, playing a crucial role in stabilizing collagen structure [53]. Acylglycines were important in regulating and detoxifying the amassing of related acyl-CoA esters and became key metabolites for diagnosing congenital metabolic defects. Additionally, butyrylglycine was measured in human urine [54].

The levels of 1-stearoyl-glycerophosphocholine (18:0), isobutyrylglycine, 1-stearoyl-2-oleoyl-glycerophosphoethanolamine (18:0/18:1), 2-butenoylglycine, 3-hydroxybutyroylglycine, and the ratio of aspartate to mannose are classified as organic acids. Organic acid metabolites primarily serve as intermediate metabolites in key metabolic pathways, encompassing fatty acid β-oxidation, protein metabolism, carbohydrate metabolism, tricarboxylic acid cycle, neurotransmitter conversion, and ketone body metabolism. The concentration of tricarboxylic acid cycle intermediates showed significant changes in most samples, indicating that hyperglycemia induces systemic stress and mitochondrial dysfunction [55]. N2,N5-Diacetylornithine has been shown to be unrelated to renal failure [56]. Furthermore, N-acetyl-isoputreanine is associated with cognitive function, potentially reflecting mechanisms involved in cognitive physiological processes [57]. Arginine to ornithine ratio worked as a biomarker for neonatal hyperargininemia [58]. Mannose receptor C (MRC2) pertained to the mannose receptor protein family and was validated to be upregulated in the kidneys of diabetic nephropathy mice [59].

Enrichment analysis identified additional metabolic pathways primarily focused on bile secretion and related metabolite processing, including the metabolism of compounds such as arginine, valine, leucine, and isoleucine. These compounds play a role in synthesizing one-carbon units as bioactive molecules.

The main origin of one-carbon units is the oxidation of amino acid carbon backbones, primarily facilitating cell progress by biosynthesizing deoxythymidine monophosphate (dTMP) and purines [60]. Early immune cell growth and activation can impact kidney function, whereas fibroblast over-proliferation in gradual deterioration is a typical pathological characteristic of DN [61]. Studies about the correlation between bile secretion pathways and DN are highly limited [62], representing a future direction for this study.

In summary, we speculate that levels of behenoylcarnitine (C22), arachidoylcarnitine (C20), and the ratio of 5-methylthioadenosine (MTA) to phosphate may contribute to the occurrence and progression of DN by increasing oxidative stress through mitochondrial dysfunction. Such an insight contributes to our comprehension of the association between serum metabolites and DN, possibly laying a good work ground for prevention strategies against this condition.

The translation of serum metabolites into clinical applications for DN involves several steps, ranging from basic research to clinical practice. This translational process aims to utilize changes in serum metabolites as biomarkers for early diagnosis, thereby monitoring the progression of DN and providing a foundation for personalized treatment. For instance, the development of serum metabolite diagnostic kits could be used for early detection and prevention of DN. Changes in metabolites can help identify different stages of DN, providing valuable information for clinical staging and individualized treatment plans based on distinct metabolic profiles. Additionally, integrating serum metabolite levels with other clinical parameters (such as urinary protein, kidney function markers, and blood glucose levels) to develop clinical decision support systems can assist physicians in making more informed treatment decisions in complex clinical settings. In conclusion, with advancements in technology, serum metabolites are poised to become a crucial tool for early diagnosis, progression monitoring, and personalized treatment of DN.

Our research presents several notable advantages. First, the large-scale sample size minimizes the influence of confounding factors on the results. Second, it provides reliable estimates of causal relationships between exposure factors and diseases, effectively addressing the issue of reverse causation often encountered in traditional observational studies. Finally, our study is the first to explore the relationship between serum metabolites and DN at the genetic level. Nevertheless, certain limitations should be acknowledged. Due to the fact that all participants are of European ancestry, caution is advised when generalizing these results to other populations. Future research should include a more diverse GWAS cohort for validation, with data that encompass factors, such as race, age, and gender.

Additionally, the available data lacked more comprehensive demographic information, such as sex and age, preventing in-depth subgroup analyses. Given the complexity and multifactorial nature of DN, our study aimed to offer a broad understanding of the underlying causalities rather than performing detailed subgroup analyses based on age, sex, or comorbidities to assess the impact of confounders on the relationship between exposure and outcome. Building upon our current findings, future studies should incorporate multivariable adjustments and stratified analyses to clarify how different subgroups (based on gender, age, or health conditions) may exhibit varying causal relationships between serum metabolites and DN. Furthermore, although this study utilized a prospective design, providing valuable insights for clinical diagnosis, further clinical validation is essential. This remains a key focus for future investigations within our research group.

## CONCLUSION

Using GWAS datasets and Mendelian Randomization (MR), our study assessed the causal relationships between serum metabolites and Diabetic Nephropathy (DN). We identified several serum metabolites, including Behenoylcarnitine (C22), arachidoylcarnitine (C20), butyrylglycine, 1-stearoyl-glycerophosphocholine (18:0), isobutyrylglycine, and 1-stearoyl-2-oleoyl-glycerophosphoethanol-amine (18:0/18:1), as genetically associated with DN. These findings offer valuable theoretical insights for the advancement of early prevention and screening strategies for DN, with significant potential clinical implications.

## Figures and Tables

**Fig. (1):**
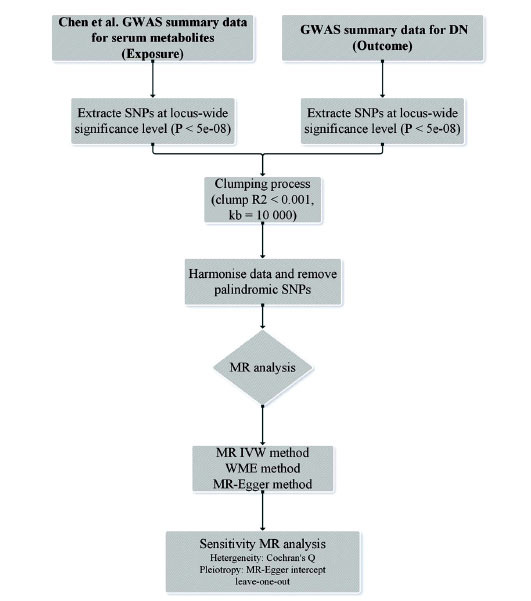
The protocol of this research.

**Fig. (8):**
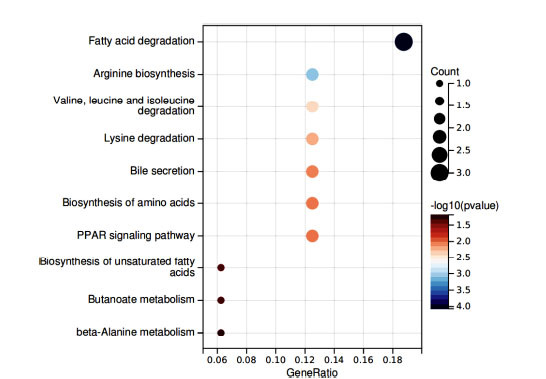
Results of pathway enrichment analysis. Each bubble size signifies the quantity of enriched genes, while the change in color signifies the importance of target gene enrichment.

## Data Availability

The produced and analysed datasets are obtained in the IEU OpenGWAS Project repository, [PERSISTENT WEB LINK, https://gwas.mrcieu.ac.uk/], accession no. is ebi-a-GCST90018832 and GCST90199621-GCST902010202.

## LIST OF ABBREVIATIONS

DN: Diabetic Nephropathy
IVs: Instrumental Variables
LD: Linkage Disequilibrium
MR: Mendelian Randomization
MRAs: Mineralocorticoid Receptor Antagonists
MTA: Methylthioadenosine
RASis: Renin-angiotensin System Inhibitors
SGLT-2is: Sodium-glucose Cotransporter-2 Inhibitors
SNPs: Single Nucleotide Polymorphisms
T2DM: Type 2 Diabetes Mellitus
