# Supplementary material for: Mendelian Randomization Study on Serum Metabolites and Diabetic Nephropathy Risk: Identifying Potential Biomarkers for Early Intervention
Source: Curr Pharm Des. 2025 May 12;32(2):143–62. doi: 10.2174/0113816128377862250429045226 (PMC12715391; doi:10.2174/0113816128377862250429045226)
Supplement: Supplementary file 1 [file CPD-32-2-143_SD1.pdf]

Supplementary Material

Mendelian Randomization Study on Serum Metabolites and Diabetic Nephropathy Risk: Identifying Potential Biomarkers for Early Intervention

Siyuan Song[1] and Jiangyi Yu[1]

[1]Jiangsu Province Hospital of Chinese Medicine, Affiliated Hospital of Nanjing University of Chinese Medicine, Nanjing, China

1873-4286/26 $65.00+.00 © 2026 Bentham Science Publishers

Table S1 Specific information on IVs applied to MR analysis.

Serum Metabolites	SNPs	Chr	EA	EAF	*Pos*	*b*	*SE*	*P*	*F*
5-hydroxylysine levels	rs144429282	3	T	0.0119106	30096963	0.387083	0.0705729	4.14E-08	30.08377062
5-hydroxylysine levels	rs3885951	15	G	0.0990508	78533575	0.578606	0.0249628	7.47E-119	537.2535221
5-hydroxylysine levels	rs58374597	15	A	0.0158799	78417591	0.610277	0.0609732	1.39E-23	100.178847
5-hydroxylysine levels	rs6495304	15	A	0.973048	78508074	0.266767	0.0475445	2.01E-08	31.48209613
Butyrylglycine levels	rs1047891	2	A	0.32329	210675783	0.297264	0.0190512	6.90E-55	243.4669203
Butyrylglycine levels	rs2014355	12	C	0.294337	120737721	0.557922	0.0183717	1.43E-202	922.2491926
Butyrylglycine levels	rs10774580	12	G	0.605206	121038620	-0.10246	0.0182646	2.03E-08	31.46939344
1-stearoyl-GPG (18:0) levels	rs1532085	15	G	0.62073	58391167	-0.112161	0.0176033	1.87E-10	40.59715171
1-stearoyl-GPG (18:0) levels	rs4149056	12	C	0.161544	21178615	0.229643	0.0228637	9.76E-24	100.8819335
1-stearoyl-GPG (18:0) levels	rs72783089	10	T	0.105114	48463526	-0.155821	0.0281216	3.01E-08	30.70237142
Isobutyrylglycine levels	rs1047891	2	A	0.315507	210675783	0.226272	0.0174476	1.84E-38	168.1861538
Isobutyrylglycine levels	rs10896190	11	C	0.561966	67638652	-0.0925512	0.0165244	2.13E-08	31.36986933
Isobutyrylglycine levels	rs1440581	4	C	0.530252	88305270	0.0940744	0.0162061	6.44E-09	33.6965847
Isobutyrylglycine levels	rs7111570	11	T	0.02861	134271204	0.380813	0.0484631	3.91E-15	61.74490826
1-stearoyl-2-oleoyl-GPE (18:0/18:1) levels	rs174541	11	C	0.353066	61798436	0.0993769	0.0161029	6.77E-10	38.08576631
1-stearoyl-2-oleoyl-GPE (18:0/18:1) levels	rs2070895	15	A	0.215848	58431740	0.23743	0.0186899	5.64E-37	161.3827938
1-stearoyl-2-oleoyl-GPE (18:0/18:1) levels	rs2414577	15	C	0.376173	58388439	0.227092	0.01579	6.71E-47	206.8423248
1-stearoyl-2-oleoyl-GPE (18:0/18:1) levels	rs964184	11	C	0.869027	116778201	-0.196287	0.0226985	5.26E-18	74.78056775
1-stearoyl-2-oleoyl-GPE (18:0/18:1) levels	rs439401	19	C	0.622459	44911194	0.0950802	0.0159868	2.72E-09	35.3717942
1-stearoyl-2-oleoyl-GPE (18:0/18:1) levels	rs12695769	3	G	0.410282	142934808	0.111596	0.0155975	8.38E-13	51.19025204
N2,N5-diacetylornithine levels	rs2947860	2	G	0.232584	73628259	0.425681	0.0176761	3.83E-128	579.9567836
N2,N5-diacetylornithine levels	rs35940501	2	T	0.126753	17415423	0.133875	0.0234817	1.19E-08	32.50423111
N2,N5-diacetylornithine levels	rs3799344	6	T	0.445273	25786765	-0.0861032	0.015636	3.66E-08	30.32405396
Behenoylcarnitine (C22) levels	rs12544463	8	G	0.269226	86468885	0.169399	0.0181917	1.26E-20	86.71118346
Behenoylcarnitine (C22) levels	rs6824723	4	G	0.192109	82641160	0.191197	0.0208226	4.22E-20	84.31255191
Behenoylcarnitine (C22) levels	rs75062282	15	C	0.0263051	85570549	0.287751	0.0516365	2.51E-08	31.05418159
Arachidoylcarnitine (C20) levels	rs140836436	22	A	0.0050821	30026468	-0.611263	0.110882	3.53E-08	30.390239
Arachidoylcarnitine (C20) levels	rs351260	5	A	0.334615	141930259	0.10656	0.0165312	1.15E-10	41.55082616
Arachidoylcarnitine (C20) levels	rs11539113	8	A	0.262092	86484947	0.141825	0.0175731	7.00E-16	65.13416105
Arachidoylcarnitine (C20) levels	rs6860806	5	G	0.552356	132304843	-0.127574	0.0160125	1.62E-15	63.4754897
2-butenoylglycine levels	rs7524467	1	G	0.322254	75728369	0.129504	0.0179485	5.38E-13	52.0607052
2-butenoylglycine levels	rs596603	11	G	0.566855	67592132	-0.138694	0.0169833	3.18E-16	66.69160737
2-butenoylglycine levels	rs1047891	2	A	0.325609	210675783	0.262111	0.0180777	1.23E-47	210.2248952
3-hydroxybutyroylglycine levels	rs1047891	2	A	0.310214	210675783	0.323747	0.0153364	6.48E-99	445.6199762
3-hydroxybutyroylglycine levels	rs596603	11	G	0.57194	67592132	-0.102531	0.0145144	1.62E-12	49.90134037
3-hydroxybutyroylglycine levels	rs7541058	1	T	0.336896	75734132	0.155071	0.0151517	1.39E-24	104.7462417
N-acetylglucosaminylasparagine levels	rs13133517	4	C	0.340305	177440833	0.145101	0.0159894	1.14E-19	82.352441
N-acetylglucosaminylasparagine levels	rs2076007	6	T	0.105759	167294271	-0.53958	0.0238664	3.59E-113	511.1376329
N-acetylglucosaminylasparagine levels	rs28929474	14	T	0.0178242	94378610	0.441029	0.0571309	1.17E-14	59.59257665

**Serum Metabolites**	**SNPs**	**Chr**	**EA**	**EAF**	***Pos***	***b***	***SE***	***P***	***F***
N-acetylglucosaminylasparagine levels	rs78357146	17	G	0.0297443	66308933	0.352596	0.0449571	4.40E-15	61.51176441
N-acetylglucosaminylasparagine levels	rs909546	6	T	0.418596	167341214	-0.128273	0.01536	6.76E-17	69.7409843
Arginine to ornithine ratio	rs791210	12	C	0.651716	120749754	-0.0986996	0.016101	8.79E-10	37.57725788
Arginine to ornithine ratio	rs17788484	6	T	0.0193313	131573218	-0.638151	0.0549643	3.65E-31	134.7986384
Arginine to ornithine ratio	rs1799918	19	C	0.365488	12891586	-0.109459	0.0157333	3.47E-12	48.40199971
Arginine to ornithine ratio	rs2608900	6	A	0.218046	131522043	0.13504	0.0186471	4.43E-13	52.4447789
Arginine to ornithine ratio	rs17157859	10	C	0.0545576	45493961	-0.185952	0.0334255	2.65E-08	30.94894738
Arginine to ornithine ratio	rs2182168	10	G	0.352905	98388596	0.0879106	0.0158989	3.21E-08	30.57372345
5-methylthioadenosine (MTA) to phosphate ratio	rs9471975	6	C	0.580273	42951484	0.0903349	0.0148411	1.15E-09	37.04921013
5-methylthioadenosine (MTA) to phosphate ratio	rs9322188	6	T	0.306899	149588355	-0.114751	0.0157678	3.40E-13	52.96277749
5-methylthioadenosine (MTA) to phosphate ratio	rs1047891	2	A	0.310004	210675783	-0.0975934	0.0159272	8.93E-10	37.54585768
5-methylthioadenosine (MTA) to phosphate ratio	rs662138	6	G	0.175365	160143444	-0.12591	0.0193196	7.16E-11	42.47410019
Aspartate to mannose ratio	rs1354034	3	C	0.607679	56815721	0.13257	0.0155541	1.55E-17	72.64416547
Aspartate to mannose ratio	rs4251689	8	T	0.471013	144515746	0.0870515	0.0152662	1.18E-08	32.51551391
Aspartate to mannose ratio	rs4665972	2	C	0.592395	27375230	-0.189749	0.0154338	9.71E-35	151.1517763

Table S2 MR estimations of serum metabolites on diabetic nephropathy risk.

Serum Metabolites	Method	*P*	*OR*	95% *CI*
5-hydroxylysine levels	Inverse variance weighted	0.004	0.743	0.606-0.910
5-hydroxylysine levels	MR Egger	0.357	0.686	0.369-1.277
5-hydroxylysine levels	Weighted median	0.024	0.760	0.600-0.964
Butyrylglycine levels	Inverse variance weighted	0.044	0.859	0.741-0.996
Butyrylglycine levels	MR Egger	0.647	0.916	0.692-1.211
Butyrylglycine levels	Weighted median	0.046	0.857	0.737-0.997
1-stearoyl-gpc (18:0) levels	Inverse variance weighted	0.034	0.685	0.483-0.971
1-stearoyl-gpc (18:0) levels	MR Egger	0.882	0.898	0.291-2.773
1-stearoyl-gpc (18:0) levels	Weighted median	0.071	0.709	0.488-1.030
Isobutyrylglycine levels	Inverse variance weighted	0.049	0.731	0.536-0.999
Isobutyrylglycine levels	MR Egger	0.217	0.538	0.272-1.064
Isobutyrylglycine levels	Weighted median	0.070	0.712	0.493-1.028
1-stearoyl-2-oleoyl-GPE (18:0/18:1) levels	Inverse variance weighted	0.035	0.793	0.639-0.984
1-stearoyl-2-oleoyl-GPE (18:0/18:1) levels	MR Egger	0.581	1.198	0.664-2.163
1-stearoyl-2-oleoyl-GPE (18:0/18:1) levels	Weighted median	0.057	0.761	0.574-1.008
N2,N5-diacetylornithine levels	Inverse variance weighted	0.049	0.652	0.426-0.997
N2,N5-diacetylornithine levels	MR Egger	0.389	0.604	0.302-1.208
N2,N5-diacetylornithine levels	Weighted median	0.078	0.667	0.425-1.046
Behenoylcarnitine (C22) levels	Inverse variance weighted	0.016	1.518	1.083-2.129
Behenoylcarnitine (C22) levels	MR Egger	0.930	0.871	0.073-10.373
Behenoylcarnitine (C22) levels	Weighted median	0.032	1.497	1.035-2.166
Arachidoylcarnitine (C20) levels	Inverse variance weighted	0.029	1.503	1.042-2.168
Arachidoylcarnitine (C20) levels	MR Egger	0.223	2.724	0.883-8.403
Arachidoylcarnitine (C20) levels	Weighted median	0.074	1.459	0.963-2.210
2-butenoylglycine levels	Inverse variance weighted	0.024	0.727	0.550-0.959
2-butenoylglycine levels	MR Egger	0.740	0.826	0.347-1.965
2-butenoylglycine levels	Weighted median	0.061	0.751	0.556-1.014
3-hydroxybutyroylglycine levels	Inverse variance weighted	0.016	0.741	0.581-0.946
3-hydroxybutyroylglycine levels	MR Egger	0.680	0.860	0.502-1.473
3-hydroxybutyroylglycine levels	Weighted median	0.043	0.772	0.600-0.992
N-acetyl-isoputreanine levels	Inverse variance weighted	0.010	0.795	0.668-0.946
N-acetyl-isoputreanine levels	MR Egger	0.276	0.817	0.607-1.101
N-acetyl-isoputreanine levels	Weighted median	0.016	0.794	0.659-0.957
Arginine to ornithine ratio	Inverse variance weighted	0.032	0.699	0.503-0.969
Arginine to ornithine ratio	MR Egger	0.255	0.590	0.271-1.286
Arginine to ornithine ratio	Weighted median	0.010	0.583	0.386-0.881
5-methylthioadenosine (MTA) to phosphate ratio	Inverse variance weighted	0.006	1.930	1.204-3.093
5-methylthioadenosine (MTA) to phosphate ratio	MR Egger	0.192	33.647	0.966-1171.413
5-methylthioadenosine (MTA) to phosphate ratio	Weighted median	0.002	2.245	1.338-3.766
Aspartate to mannose ratio	Inverse variance weighted	0.035	0.695	0.495-0.975
Aspartate to mannose ratio	MR Egger	0.807	1.198	0.386-3.722
Aspartate to mannose ratio	Weighted median	0.107	0.748	0.526-1.065

Table S3 Sensitivity analysis.

Serum Metabolites	Method	Cochran Q	Q value	egger_intercept	*P* value
5-hydroxylysine levels	MR Egger	2.070	0.355	0.039	0.816
5-hydroxylysine levels	Inverse variance weighted	2.140	0.543		
Butyrylglycine levels	MR Egger	0.416	0.519	-0.026	0.692
Butyrylglycine levels	Inverse variance weighted	0.693	0.707		
1-stearoyl-gpc (18:0) levels	MR Egger	0.012	0.911	-0.047	0.708
1-stearoyl-gpc (18:0) levels	Inverse variance weighted	0.257	0.880		
Isobutyrylglycine levels	MR Egger	0.852	0.653	0.053	0.426
Isobutyrylglycine levels	Inverse variance weighted	0.836	0.607		
1-stearoyl-2-oleoyl-GPE (18:0/18:1) levels	MR Egger	2.190	0.701	-0.075	0.215
1-stearoyl-2-oleoyl-GPE (18:0/18:1) levels	Inverse variance weighted	4.360	0.499		
N2,N5-diacetylornithine levels	MR Egger	0.716	0.397	0.016	0.831
N2,N5-diacetylornithine levels	Inverse variance weighted	0.791	0.673		
Behenoylcarnitine (C22) levels	MR Egger	0.158	0.691	0.105	0.734
Behenoylcarnitine (C22) levels	Inverse variance weighted	0.355	0.837		
Arachidoylcarnitine (C20) levels	MR Egger	0.345	0.842	-0.086	0.388
Arachidoylcarnitine (C20) levels	Inverse variance weighted	1.544	0.672		
2-butenoylglycine levels	MR Egger	0.502	0.479	-0.025	0.811
2-butenoylglycine levels	Inverse variance weighted	0.595	0.703		
3-hydroxybutyroylglycine levels	MR Egger	0.723	0.395	-0.035	0.652
3-hydroxybutyroylglycine levels	Inverse variance weighted	1.093	0.579		
N-acetyl-isoputreanine levels	MR Egger	1.460	0.691	-0.01	0.834
N-acetyl-isoputreanine levels	Inverse variance weighted	1.520	0.824		
Arginine to ornithine ratio	MR Egger	3.720	0.445	0.024	0.665
Arginine to ornithine ratio	Inverse variance weighted	3.940	0.559		
5-methylthioadenosine (MTA) to phosphate ratio	MR Egger	0.883	0.643	-0.302	0.253
5-methylthioadenosine (MTA) to phosphate ratio	Inverse variance weighted	3.413	0.332		
Aspartate to mannose ratio	MR Egger	0.318	0.573	-0.081	0.504
Aspartate to mannose ratio	Inverse variance weighted	1.294	0.524		

Table S4 SNPs identified converted into Gene IDs.

id	chr	start	end	strand	gene_ids	gene_names	3_prime_utr_variant	nmd_transcript_variant	intron_variant	mis-sense_variant	non_coding_tran-script_exon_variant	non_coding_tran-script_variant	stop_gained	synon-ymous_variant
rs144429282		-1	-1				0	0	0	0	0	0	0	0
rs3885951	15	78533575	78533575	+	ENSG00000188266	HYKK	0	0	2	2	0	0	0	0
rs58374597		-1	-1				0	0	0	0	0	0	0	0
rs6495304	15	78508074	78508074	+	ENSG00000188266	HYKK	0	3	12	0	0	0	0	0
rs1047891	2	210675783	210675783	+	ENSG00000021826	CPS1	1	1	0	7	4	0	0	0
rs2014355	12	120737721	120737721	+	ENSG00000122971	ACADS	0	0	2	0	1	0	0	0
rs10774580	12	121038620	121038620	+	ENSG00000135114	OASL	0	6	18	0	0	0	0	0
rs1532085	15	58391167	58391167	+	ENSG00000128918	ALDH1A2	0	0	4	0	0	2	0	0
rs4149056	12	21178615	21178615	+	ENSG00000134538	SLCO1B1	0	0	0	4	0	0	0	0
rs72783089	10	48463526	48463526	+	ENSG00000128805	ARHGAP22	0	1	10	0	0	3	0	0
rs10896190	11	67638652	67638652	+	ENSG00000167800	TBX10	0	0	1	0	0	0	0	0
rs1440581	4	88305270	88305270	+	ENSG00000246375	PPM1K-DT	0	0	24	0	0	24	0	0
rs7111570		-1	-1				0	0	0	0	0	0	0	0
rs174541	11	61798436	61798436	+	ENSG00000134824, ENSG00000289268	FADS2, ENSG00000289268	0	0	1	0	1	1	0	0
rs2070895	15	58431740	58431740	+	ENSG00000128918, ENSG00000166035	ALDH1A2, LIPC	0	0	10	0	0	4	0	0
rs2414577	15	58388439	58388439	+	ENSG00000128918	ALDH1A2	0	0	4	0	0	2	0	0
rs964184	11	116778201	116778201	+	ENSG00000109917	ZPR1	3	0	0	0	0	0	0	0
rs439401	19	44911194	44911194	+	ENSG00000280087	ENSG00000280087	0	0	0	0	1	0	0	0
rs12695769	3	142934808	142934808	+	ENSG00000243818	ENSG00000243818	0	0	9	0	1	9	0	0
rs2947860		-1	-1				0	0	0	0	0	0	0	0
rs35940501		-1	-1				0	0	0	0	0	0	0	0
rs3799344	6	25786765	25786765	+	ENSG00000124568	SLC17A1	0	3	6	0	0	0	0	0
rs12544463	8	86468885	86468885	+	ENSG00000123124, ENSG00000176623	WWP1, RMDN1	0	0	3	0	0	0	0	0
rs6824723	4	82641160	82641160	+	ENSG00000145284	SCD5	0	0	1	0	0	0	0	0
rs75062282	15	85570549	85570549	+	ENSG00000170776	AKAP13	0	0	5	0	0	0	0	0
rs140836436	22	30026468	30026468	+	ENSG00000100330, ENSG00000227117, ENSG00000279159	MTMR3, HORMAD2-AS1, ENSG00000279159	4	0	2	0	1	2	0	0
rs351260	5	141930259	141930259	+	ENSG00000081791	DELE1	0	0	0	3	1	0	0	0
rs11539113	8	86484947	86484947	+	ENSG00000176623	RMDN1	4	4	6	0	2	2	5	5
rs6860806	5	132304843	132304843	+	ENSG00000197208	SLC22A4	0	0	4	0	0	2	0	0
rs7524467	1	75728369	75728369	+	ENSG00000117054	ACADM	0	64	108	0	2	26	0	0
rs596603		-1	-1				0	0	0	0	0	0	0	0
rs7541058	1	75734132	75734132	+	ENSG00000117054	ACADM	0	32	50	0	6	9	0	0
rs13133517	4	177440833	177440833	+	ENSG00000038002	AGA	0	0	4	0	0	3	0	0
rs2076007	6	167294271	167294271	+	ENSG00000112494	UNC93A	0	0	4	0	0	1	0	0
rs28929474	14	94378610	94378610	+	ENSG00000197249	SERPINA1	2	2	0	20	0	0	0	0
rs78357146	17	66308933	66308933	+	ENSG00000154229	PRKCA	0	2	4	0	0	1	0	0

id	chr	start	end	strand	gene_ids	gene_ names	3_prime _utr_ variant	nmd_ transcript_ variant	intron_ variant	mis- sense_ variant	non_ coding_ tran- script_ exon_ variant	non_ coding_ tran- script_ variant	stop_ gained	synon- ymous_ variant
rs909546	6	167341214	167341214	+	ENSG00000120440	TTLL2	0	2	0	3	0	0	0	3
rs791210	12	120749754	120749754	+	ENSG00000255946	ENSG000002 55946	0	0	2	0	0	2	0	0
rs17788484	6	131573218	131573218	+	ENSG00000118520	ARG1	0	1	3	0	1	1	0	0
rs1799918	19	12891586	12891586	+	ENSG00000105607	GCDH	0	2	10	1	2	4	0	0
rs2608900	6	131522043	131522043	+	ENSG00000118520	ARG1	0	1	3	0	0	1	0	0
rs17157859	10	45493961	45493961	+	ENSG00000165406	MARCHF8	0	0	5	0	0	1	0	0
rs2182168	10	98388596	98388596	+	ENSG00000119943	PYROXD2	0	0	4	0	0	2	0	0
rs9471975		-1	-1				0	0	0	0	0	0	0	0
rs9322188	6	149588355	149588355	+	ENSG00000055211, ENSG00000281021	GINM1, ENSG000002 81021	0	2	6	0	0	2	0	0
rs662138	6	160143444	160143444	+	ENSG00000175003	SLC22A1	0	2	5	0	0	0	0	0
rs1354034	3	56815721	56815721	+	ENSG00000163947	ARHGEF3	0	2	10	0	0	3	0	0
rs4251689	8	144515746	144515746	+	ENSG00000160957	RECQL4	0	0	4	0	0	0	0	0
rs4665972	2	27375230	27375230	+	ENSG00000115234	SNX17	0	3	7	0	0	2	0	0
